# Virtual Network Embedding for Multi-Domain Heterogeneous Converged Optical Networks: Issues and Challenges

**DOI:** 10.3390/s20092655

**Published:** 2020-05-06

**Authors:** Yue Zong, Chuan Feng, Yingying Guan, Yejun Liu, Lei Guo

**Affiliations:** 1 School of Computer Science and Engineering, Northeastern University, Shenyang 110819, China; fcyaokuaile@163.com (C.F.); guanyingying@stumail.neu.edu.cn (Y.G.); 2 School of Communication and Information Engineering, Chongqing University of Posts and Telecommunications, Chongqing 400065, China; yjliu@cqupt.edu.cn (Y.L.); guolei@cqupt.edu.cn (L.G.)

**Keywords:** virtual network embedding, converged optical networks, network slicing, machine learning, software-defined network

## Abstract

The emerging 5G applications and the connectivity of billions of devices have driven the investigation of multi-domain heterogeneous converged optical networks. To support emerging applications with their diverse quality of service requirements, network slicing has been proposed as a promising technology. Network virtualization is an enabler for network slicing, where the physical network can be partitioned into different configurable slices in the multi-domain heterogeneous converged optical networks. An efficient resource allocation mechanism for multiple virtual networks in network virtualization is one of the main challenges referred as virtual network embedding (VNE). This paper is a survey on the state-of-the-art works for the VNE problem towards multi-domain heterogeneous converged optical networks, providing the discussion on future research issues and challenges. In this paper, we describe VNE in multi-domain heterogeneous converged optical networks with enabling network orchestration technologies and analyze the literature about VNE algorithms with various network considerations for each network domain. The basic VNE problem with various motivations and performance metrics for general scenarios is discussed. A VNE algorithm taxonomy is presented and discussed by classifying the major VNE algorithms into three categories according to existing literature. We analyze and compare the attributes of algorithms such as node and link embedding methods, objectives, and network architecture, which can give a selection or baseline for future work of VNE. Finally, we explore some broader perspectives in future research issues and challenges on 5G scenario, field trail deployment, and machine learning-based algorithms.

## 1. Introduction

The exponential growth of the emerging of dynamic applications and the billions of devices in the Internet of Things (IoT) with sensing, computing, and communication capabilities have driven the investigation of network architecture. Current network architectures fail to address the diverse performance requirements in terms of latency, scalability, availability, and reliability [1]. To overcome the issues and support more heterogeneous applications, network slicing is considered as a promising technology formed by partitioning or combining a set of network resources, and abstracting it to users [2,3]. Network virtualization and orchestration are key processes for network slicing, where software-defined network (SDN) and network function virtualization (NFV) are the key enabling technologies for network orchestration [4,5]. In addition, SDN can manage and deploy the service requirements automatically by decoupling the transmitting layer and control layer [6,7]. It is specified to leverage the benefits of network virtualization to allow high flexibility among various mobile and IoT services in multi-domain heterogeneous converged optical networks.

Infrastructure provider (InP) and service provider (SP) have been decoupled in the network virtualization environment to enable multiple virtual networks (VNs) coexisting and sharing substrate resources (e.g., node computing resources and link resources) [8,9]. Efficient resource allocation for both virtual nodes and links is one of the major challenges which refers as virtual network embedding (VNE) [10]. Many existing works have solved the VNE resource allocation problem by integer linear program (ILP) [11,12,13]. Efficient VNE algorithms have been proposed in many works to improve the performance [14,15], which include two-stage algorithms by efficient node ranking and link assignment method, coordinated VNE approaches, and machine learning (ML)-based algorithms. Many existing works have focused on specific domain network architecture of heterogeneous converged optical networks for the VNE approaches, such as wireless network, fiber-wireless (FiWi) access network, and optical data center network (ODCN).

Existing surveys and literature have considered various details and network features for VNE under different network scenarios (e.g., elastic optical network) [16,17,18]. In [16], the authors have focused on algorithmic aspects for VNE for cloud networks. However, these surveys have not focused on VNE for multi-domain heterogeneous converged optical networks and the future research issues on implementation and intelligent algorithms. In this paper, the representative references in the latest popular top journals and conferences about VNE and network slicing are discussed. Furthermore, multi-domain heterogeneous converged optical network architecture has been described and the differences among them are discussed, e.g., radio resource for wireless channel, spectrum characteristics for optical network, and various substrate nodes. Thereby, the characteristics of specific single domain network for VNE have been discussed. We provide a brief survey of the basic VNE problem formulas and a taxonomy of VNE approaches on existing works. Issues and challenges have been discussed for the road on VNE in the future.

The organization of the paper is as follows. VNE in multi-domain heterogeneous converged optical networks and key enabling technologies are discussed in Section 2. In Section 3, the basic VNE problem and major metrics are presented. In Section 4, we give a VNE algorithm taxonomy for existing works. The future issues and challenges on the road of VNE are discussed in Section 5. Finally, we conclude the paper in Section 6.

## 2. Virtual Network Embedding in Multi-Domain Heterogeneous Converged Optical Networks

**Figure 1:**
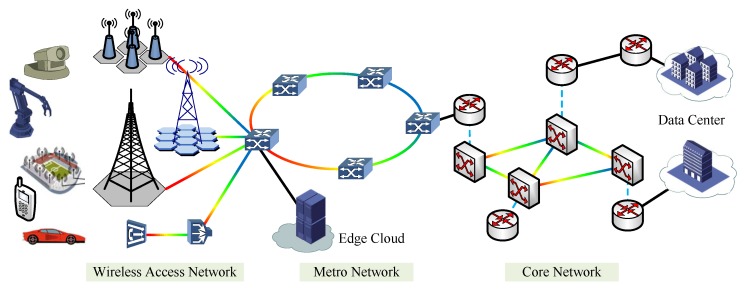
Architecture of multi-domain heterogeneous converged optical networks.

For emerging enhanced mobile broadband (eMBB), massive machine communications, and ultra-reliable and ultra-low latency communications 5G scenarios, network slicing as a promising technology can guarantee the requirements and efficient resource utilization. The diagram of multi-domain heterogeneous converged optical network architecture in Figure 1 is composed of wireless access network domain, metro network domain, core network domain, and edge computing/data center domain. Wireless access networks have been architectured to support a number of diverse vertical applications of end users and converged the requirements into metro and core networks. Edge computing and data centers provide computing capacity to guarantee the implementation of VNE. Many 5G research works and demonstration projects (e.g., 5GNORMA, 5GEx, 5GinFIRE, and 5G!Pagoda) have addressed the realization of 5G slicing mainly on wireless domain through the combination of key enabling technologies of SDN and NFV [19]. Thereby, SDN and NFV are described in the following as key enabling technologies to provide and guarantee the deployment and implementation of VNE. Although there exist few researches about VNE for 5G multi-domain networks, many of the existing works have focused on VNE for single domain. We review the VNE in wireless network, FiWi access network, and optical network single domain in the following.

### 2.1. Key Enabling Technologies

VNE implementation, resource allocation, and scheduling are key points in multi-domain heterogeneous networks. SDN and NFV have been proposed as key enabling technologies to achieve network slicing orchestration for many 5G researches and demonstration projects. SDN can decouple the infrastructure layer and the control layer, which is considered a key enabling technology to implement network virtualization (e.g., deployment VNE algorithms) and manage services for network operators [20,21,22]. The SDN controller enables configuring network remotely to enhance the network flexibility and service provisioning, which can provide a closer tie between application requirements and the combination of resources (e.g., optical transport network, IP layer, computing, and storage) [23]. SDN is an ideal platform for implementation of network virtualization since it can flexibly offer end-to-end network slices according to the requirements of different applications through hypervisors such as Flowvisor, OpenVirteX, FlowN, and AutoVFlow [24].

NFV plays an important role in the realization of virtual network functions and network services by decoupling network functions from the dedicated physical devices, implementing them as software on industrial standard high volume servers. Service chaining composed of virtualized network functions can make both data and control plane functions flexible so that the traffic of certain users or applications only traverses a particular set of functions [25]. Virtual network functions placement can be regarded as a special VNE issue. Thereby, the complexity of VNE problem for network management and deployment in 5G multi-domain network architecture has increased, especially in the context of large number of VN requests. Management and orchestration (MANO) framework is leveraged as a critical automatically solution to manage and orchestrate network virtualization [26,27].

### 2.2. Wireless Network

**Figure 2:**
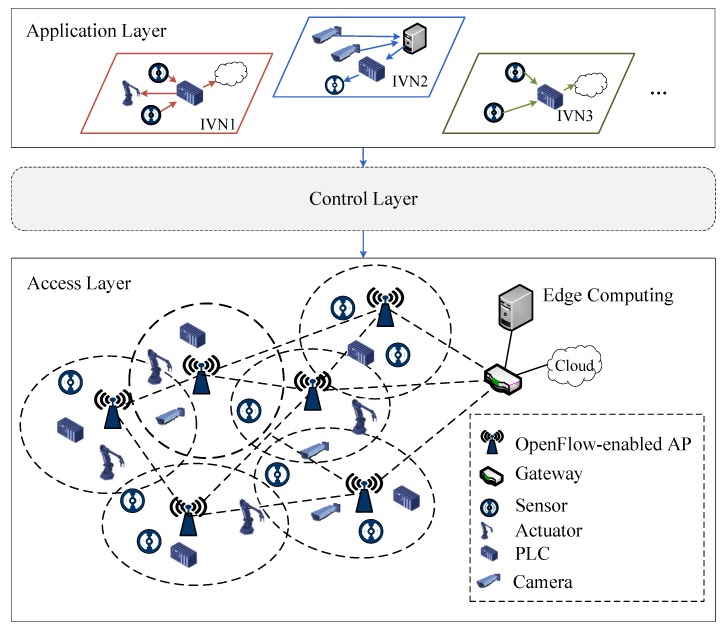
Illustration of slice-based virtualization for industrial wireless networks (IWNs).

Due to the development of emerging 5G applications (e.g., IoT and Internet of Vehicles (IoV)), the VNE problem in wireless network domain of 5G has gained more attention due to the growing popularity of 5G applications. The implementation and efficient slicing of wireless domain are essential to provide services for users as it is closed to the massive users side in 5G networks. The concept of the cloud-radio access network (C-RAN) has been proposed to decouple digital units (DUs) and radio units (RUs) of base stations (BSs) and centralize DUs into central offices [28]. C-RAN is supposed to increase the capacity by 1000x to handle the growing number of connected devices and increasing data rates, which can ease the implementation of advanced radio coordination techniques, e.g., coordinated multi-point (CoMP) Transmission/Reception. In addition, the revolution of IoT is reshaping the modern industrial systems, where industrial wireless networks (IWNs) refer to the pervasive deployment of devices with sensing, processing, and connecting capabilities [24]. The example of VNE in IWNs is shown in Figure 2, where massive devices deployed in the access layer perform monitoring and controlling. Those OpenFlow-enabled access points (APs) with mesh topology compose the data plane for packet accessing and transferring to edge and cloud computing. The substrate network is controlled by the controller of the control layer to satisfy the industrial virtual network in the application layer.

According to the European Telecommunications Standards Institute (ETSI) [29], mobile edge computing (MEC) is defined as “Mobile edge computing provides an IT service environment and cloud computing capabilities at the edge of the mobile network, within the radio access network (RAN) and in close proximity to mobile subscribers.” To extend cloud computing services to the edge of networks leveraging mobile base stations, MEC is an emergent architecture which can be applied to mobile, wireless, and wireline scenarios, using software and hardware platforms, located at the network edge in the vicinity of end users [30].

Wireless sensor networks (WSN) are regarded as the basic constituents of IoT that can facilitate the interaction of users (humans or machines) with their environment and react to real-world events. To create large-scale sensor platforms, WSN virtualization is envisioned as an important technology to satisfy efficient usage of network resources. The authors of [31] have analyzed the importance and approaches for sensor node-level virtualization and network-level virtualization of WSN. To facilitate the QoS provisioning for different applications with strict demands on latency and reliability, an application-driven virtual network embedding scheme has been proposed for flexible network resource allocation of industrial WSNs [32].

In addition, many unique characteristics of wireless networks need to be considered for VNE [32,33,34,35]. Node mapping is only partially deterministic, as the AP is simply selected according to the location of users, but gateway (GW) has to be determined based on throughput optimization [34]. Mobility for node (re-)mapping has been considered in [35]. Due to the broadcast nature of wireless channels, link mapping needs to consider the specific multiple access mechanisms. The authors of [32] propose an approach based on anypath routing technique to reduce the resources consumed by re-transmission.

### 2.3. Fiber-Wireless Access Network

The FiWi access network is a network converged by wireless and optical components, and is an essential part of 5G networks as it guarantees the 5G service requirements and converges them into the core network. It gains more popularity for its advantages of high capacity, long distance, and flexible access ability [36,37,38]. Network virtualization for FiWi is required to overcome the bottleneck of joint wireless and optical resource allocation.

**Figure 3:**
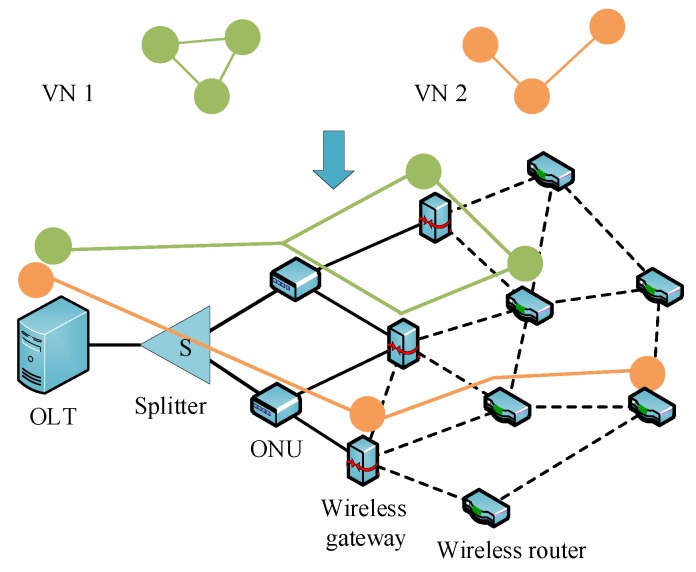
Illustration of virtual network embedding (VNE) in fiber-wireless (FiWi) access network.

Additional network characteristics of FiWi need to be considered, where the substrate nodes include optical line terminal (OLT), optical network unit (ONU), wireless router, and wireless gateway. The substrate links are composed of fiber link, cable, and wireless link. Additional link features are supposed to be considered for link embedding, e.g., the channels of wireless radio interfaces. The illustration of VNE in FiWi access network [39] is shown in Figure 3. Efficient resource management for both optical and wireless resources of the SDN/NFV-based converged network has been discussed in [40] to guarantee the specific delay and bandwidth requirements of the multiple services of network slices. Furthermore, to centralize control and allocate network and computing resources of converging edge computing over FiWi network, the authors of [41] propose two VNE algorithms to obtain higher revenue and profit ratio.

### 2.4. Optical Network

Optical network is the fundamental part in 5G multi-domain heterogeneous network to ensure the high bandwidth and low latency transmission. Many existing works have proposed efficient VNE allocation schemes from various aspects to guarantee the converged massive 5G services performance requirements for network virtualization [42,43,44,45]. A novel dynamic VNE approach based on an auxiliary graph is proposed to improve network utilization and performance by adjusting the weights of the edges of the auxiliary graph on fixed-grid DWDM network [43]. The proposed VNE algorithms for migration in [44] have improved network utilization and energy consumption efficiently.

To facilitate the flexible allocation of the fiber spectrum, elastic optical network (EON) is an emerging technology by leveraging finer-grained channel spacing, tunable modulation formats and forward error correction overheads, and baud-rate assignment [46]. In [11,14,45], the authors have proposed efficient approaches to solve VNE in EON for network slicing to guarantee the service requirements. Spatial resources of optical network are also considered in some works [47,48,49], which refer to fiber cores or modes in multi-core fibers or multi-mode fibers, or even single-mode fiber bundles. The proposed genetic algorithm in [48] has obtained the optimal VNE schemes with core allocation to efficiently by designing tailor-made encoding scheme, crossover, and mutation operators. Some additional constraints for VNE should be considered for optical network domain such as spectrum continuity, spectrum contiguity, and physical layer impairments [50,51,52].

**Figure 4:**
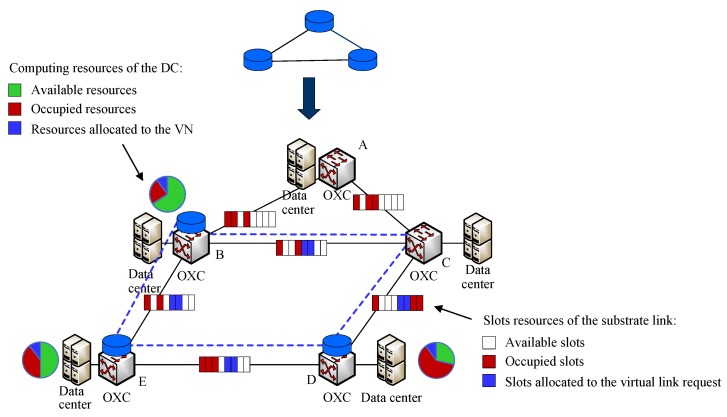
Illustration of VNE in inter-optical data center networks (ODCNs).

Data centers (DCs) have become an efficient and promising infrastructure to provide data storage and computing capacity. Geographical distribution data center networks connected with optical network guarantee the requirements of 5G network services and applications (e.g., video streaming) [53,54,55]. Furthermore, network virtualization in ODCNs can be classified into intra- [17,18] and inter-ODCNs [16,56,57,58]. Three provisioning schemes have been proposed in [59] by constructing a virtual auxiliary graph that decomposes the physical infrastructure into several layered graphs, according to the spectrum slot requirements of a virtual optical network request. Network services deployment and orchestration for network slice in inter-ODCNs have been developed in [60]. OpenStack-based orchestrator deploys the VMs for IT requirements by the path computation engine and contacts with the OpenDaylight SDN controller to guarantee the network configuration. The illustration of VNE in inter-ODCN and the network architecture are shown in Figure 4.

## 3. Virtual Network Embedding Problem

This section introduces the substrate network, virtual network, and general VNE problem description and formulation with restrictions for resource allocation. Furthermore, the major objectives and efficiency metrics are formulated to evaluate the performance of VNE problem for resource allocation.

### 3.1. Substrate Network

Similar to the work in [13], the substrate network is described by an undirected graph $G^{S}=\left(N^{S},E^{S},A_{N}^{S},A_{E}^{S}\right)$, where $N^{S}$ is the set of substrate nodes and $E^{S}$ refers to the set of substrate links. Substrate nodes and links are associated with their attributes, denoted by $A_{N}^{S}$ and $A_{E}^{S}$, respectively. For each substrate node $n\in N^{S}$, the node attributes usually consider CPU capacity $C_{n}^{S}$ and location $Loc_{n}^{S}$. For substrate link $e^{S}\left(m,n\right)$ between the substrate node *m* and *n*, the typical attribute is bandwidth capacity $b_{e}^{S}$ [61] and wireless channel [37]. Additional link constraints (i.e., wavelength, spectrum continuity, and spectrum contiguity) need to be considered when the substrate link is optical fiber [51,52]. An important issue has to be taken into account while the actual effect of users mobility for wireless networks [62].

### 3.2. Virtual Network

The undirected graph, $G^{V}=\left(N^{V},E^{V},A_{N}^{V},A_{E}^{V}\right)$, describes the set of virtual network requests [63], where $N^{V}$ and $E^{V}$ refer to the sets of virtual nodes and links, respectively. The network topology, $\left(N^{V},E^{V}\right)$, is a logical network that should be configured as a sub-network of the substrate network. Typically, the substrate network should satisfy the attributes associated with virtual nodes and links described as $A_{N}^{V}$ and $A_{E}^{V}$, respectively. In addition, for the virtual node $v\in N_{r}^{V}$ of the $r^{th}$ VN, the requested node attributes for embedding are CPU capacity request $c_{r}^{v}$, the location $Loc_{r}^{v}$, and maximum location distance $\rho_{r}^{v}$. For each virtual link $e_{r}\left(v,u\right)\in E_{r}^{V}$, bandwidth requirement, $B_{e_{r}}^{V}$, is the major considered virtual link attribute.

### 3.3. Virtual Network Embedding

**Figure 5:**
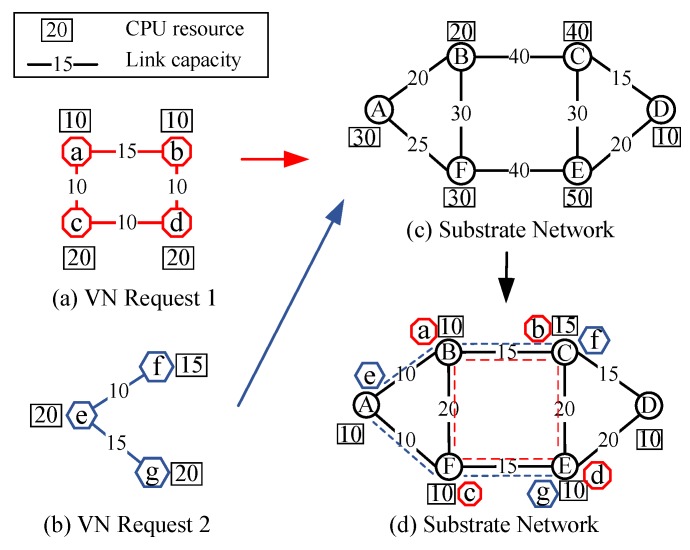
Example of VN embedding solution.

The problem of mapping virtual network to substrate network can be defined as a mapping *M*: $G^{V}\left(N^{V},E^{V}\right)$→$G^{S}\left(N^{S},E^{S}\right)$, from $G^{V}$ to a subset of $G^{S}$ [11,14,15]. Figure 5d shows the example of VN embedding solution for virtual network requests in Figure 5a,b embedded in initial substrate network shown in Figure 5c. The numbers over the links represent the link capacity and the numbers in rectangles represent CPU resource of virtual network and substrate network. VNE can be decomposed into two steps:

1. Virtual Node Embedding (VNoE): $f:N_{r}^{V}\to N^{S}$.
  Virtual nodes need to be embedded to different substrate nodes that satisfy the node resource and location constraints, which are described by Equations (1)–(4), where $\delta_{n}^{v,r}\in$ {0,1}. If virtual node *v* of $r^{th}$ VN is embedded into substrate node *n*, $\delta_{n}^{v,r}=1$. Equation (1) guarantees that all virtual nodes that are accommodated by the substrate node *n* cannot exceed the total substrate computing resource. Each virtual node *v* can only play host once to a unique substrate node shown in Equation (2). Each substrate node *n* can only host one virtual node of the same VN request described by Equation (3). The distance constraint for each virtual node is described by Equation (4), where dis(·) refers the distance between the locations of substrate node *n* and virtual node *v*.
  (1) $$\sum_{r\in R}\sum_{v\in N_{r}^{V}}c_{r}^{v}\cdot \delta_{n}^{v,r}\leq C_{n}^{S}\;\forall n\in N^{S}$$
  (2) $$\sum_{v\in N_{r}^{V}}\delta_{n}^{v,r}\leq 1\;\forall n\in N^{S},\;\forall r\in R$$
  (3) $$\sum_{n\in N^{S}}\delta_{n}^{v,r}=1\;\forall v\in N_{r}^{V},\;\forall r\in R$$
  (4) $$dis\left(Loc_{r}^{v},Loc_{n}^{S}\right)\leq \rho_{r}^{v}\;\forall n\in N^{S},\forall r\in R,\;v\in N_{r}^{V}$$
2. Virtual Link Embedding (VLiE): $f:E_{r}^{V}\to E^{S}$.
  Virtual links embedded to loop-free paths on the substrate network that satisfy the link bandwidth resource requirements and the total virtual link requirements cannot exceed the bandwidth resource of substrate link $e^{S}\left(m,n\right)$, as shown in Equation (5). Binary variable $f(e_{r}\left(v,u\right),e^{S}\left(m,n\right))$ equals 1, if substrate link $e^{S}$ is embedded by virtual link $e_{r}$. Flow conservation constraint is shown in Equation (6). According to features of substrate links, additional link constraints should be considered, i.e., optical wavelength, spectrum continuity in EON [50,51,52], and wireless channel, expected anypath transmission time of anypath [24].
  (5) $$\sum_{r\in R}\sum_{e_{r}\in E_{r}^{V}}f\left(e_{r}\left(v,u\right),e^{S}\left(m,n\right)\right)\cdot B_{e_{r}}^{V}\leq b_{e}^{S},\;\forall e^{S}\in E^{S}$$
  (6) $$\sum_{n\in N^{S}}f\left(e_{r}\left(v,u\right),e^{S}\left(m,n\right)\right)-\sum_{n\in N^{S}}f\left(e_{r}\left(v,u\right),e^{S}\left(n,m\right)\right)=\delta_{m}^{v,r}-\delta_{m}^{u,r},\;\forall e_{r}\left(v,u\right)\in E_{r}^{V},\forall r\in R$$

### 3.4. Main Objectives and Metrics

The VNE problem needs to evaluate the performance for VNE approaches and satisfy the diverse 5G service requirements. Thereby, one or some metrics are considered as objectives according to the motivation of literatures. In this section, the major objectives and metrics are described and analyzed.

#### 3.4.1. Profit

Similar to work in [64,65], the revenue of serving a VN request is defined by summing up the required CPU and bandwidth resource for VN requests as shown in Equation (7), where α and β are the weights. Although revenue is the metric that InP will gain by accepting VN requests, it is not very considerable without knowing the cost of InP. The cost of VNE is defined by summing up all CPU and bandwidth resources of the substrate network resources allocated for VN requests. The embedding cost for VN request *r* is described by Equation (8), where $\alpha_{c}$ and $\beta_{c}$ are the weights for CPU and bandwidth costs, respectively. In addition, the whole profit for InP for serving all the VN requests is defined by Equation (9). The revenue/cost ratio described by Equation (10) indicates the percentage between revenue and cost, where it also can reflect the profit for InP.

(7) $$Revenue\left(G_{r}^{V}\right)=\alpha \cdot \sum_{v\in N_{r}^{V}}c_{r}^{v}+\beta \cdot \sum_{e_{r}\in E_{r}^{V}}B_{e_{r}}^{V}$$

(8) $$Cost\left(G_{r}^{V}\right)=\alpha_{c}\cdot \sum_{v\in N_{r}^{V}}c_{r}^{v}+\beta_{c}\cdot \sum_{e_{r}\in E_{r}^{V}}\sum_{e^{S}\in E^{S}}f\left(e_{r}\left(v,u\right),e^{S}\left(m,n\right)\right)\cdot B_{e_{r}}^{V}$$

(9) $$Prf\left(G^{V}\right)=\sum_{r\in R}Revenue(G_{r}^{V})-\sum_{r\in R}Cost\left(G_{r}^{V}\right)$$

(10) $$Revenue/Cost\;Ratio=\frac{\sum_{r\in R}Revenue(G_{r}^{V})}{\sum_{r\in R}Cost\left(G_{r}^{V}\right)}$$

#### 3.4.2. Acceptance Ratio

The acceptance ratio is also related with profit, as the revenue is calculated if the VN request is accepted [64]. As described in Equation (11), it measures the number of VN requests which are completely embedded, where $Acc_{r}^{V}\in \left\{0,1\right\}$ refers VN request *r* is accepted or not. For some online problem, usually blocking ratio is considered, where BlockingRatio=1−AcceptanceRatio.

(11) $$Acceptance\;\;Ratio=\frac{\sum_{G_{r}^{V}\in G^{V}}\;Acc_{r}^{V}}{|G^{V}|}$$

#### 3.4.3. Resource Utilization

Resource utilization is defined by summing the occupied substrate resources (node and link) for the embedded VN requests divided by the total amount of resources. This metric can take into account the resource usage. Node resource utilization $RU_{Com}$ and link resource utilization $RU_{Link}$ are shown in Equations (12) and (13) by summing the requirements of the embedded node or link for VN requests divided by the total node or link capacity of the substrate network [66]. For some online scenarios, the lifetime of VN requests is considered.

(12) $$RU_{Com}=\frac{\sum_{n\in N^{S}}\sum_{r\in R}\sum_{v\in N_{r}^{V}}\;c_{r}^{v}\cdot \delta_{n}^{v,r}}{\sum_{n\in N^{S}}\;C_{n}^{S}}$$

(13) $$RU_{Link}=\frac{\sum_{e^{S}\in E^{S}}\sum_{r\in R}\sum_{e_{r}\in E_{r}^{V}}f\left(e_{r}\left(v,u\right),e^{S}\left(m,n\right)\right)\cdot B_{e_{r}}^{V}}{\sum_{e^{S}\in E^{S}}\;b_{e}^{S}}$$

#### 3.4.4. Latency

As the emerging 5G services (e.g., IoV) have strict latency requirements, the VNE problem must guarantee these targets [67]. In the existing literature, latency requirements have been modeled as constraints applied to the virtual links of VN (i.e., each virtual link is mapped to satisfy a given latency target) [45]. The authors of [68,69,70] focus on latency-aware algorithms by considering the time (e.g., propagation time or delay) from one embedded substrate node to another to satisfy the requirements of virtual links. For simplicity, path length is used to represent the latency metric in [69], which sums up the length of substrate links where a virtual link request is embedded. In connection with the longer length of corresponding path, more resources are consumed in substrate network and users will suffer longer latency.

Especially for 5G end users that require ultra-low latency services (e.g., video broadcast service, gaming service), link latency should obtain more consideration. The authors of [71] have formulated the end-to-end delay in a fronthaul network as $D_{e2e}=D_{proc}+D_{prop}+D_{link}+D_{queue}$. The total processing delay $D_{proc}$ is a fixed value required to forward a packet. $D_{prop}$ is the propagation delay, which is determined by the fiber length. The serialization delay $D_{link}$ is proportional to the frame size and inversely proportional to the link bandwidth capacity. Queuing delay $D_{queue}$ is caused by the competition among fronthaul packets. The authors of [45] focus on latency model in EON, where the latency of lightpath is shown as $L_{p}=L_{n}+len\left(p\right)L_{prop}+n_{amp}L_{amp}+\left(|p|+1\right)L_{roadm}$. $L_{n}$ means the latency at terminal node considering FEC modules and transponders. Propagation delay $len\left(p\right)L_{prop}$ is the major latency contribution for a lightpath, which $L_{prop}$ amounts to≈4.9μs per kilometer of fiber and len(p) is the physical length of lightpath. The latency of amplifiers $n_{amp}L_{amp}$ is considered, where $n_{amp}$ is the number of amplifiers on a lightpath *p*. The latency of component reconfigurable optical add-drop multiplexer is shown by $L_{roadm}$ and |p| is the number of substrate optical links on the lightpath. The differences between the two equations mentioned above are based on the network components of substrate links.

#### 3.4.5. Energy Efficiency

Energy consumption of network infrastructures in network virtualization has been focused due to the rising energy costs and ecological awareness. Without compromising the network performance (e.g., InPs), switching off or sleeping power-consuming elements by consolidating requests is considered as the primary approaches to minimize the energy consumption in [66,69,72]. Various network components in network architecture (e.g., servers, routers, and transponders) are considered in [73,74,75]. In general, the ratio between running nodes and the total number of substrate nodes is taken into account as a performance metric. The authors of [76] have considered migration to re-optimize the energy consumption with consideration of interruption time and bandwidth waste of migration.

#### 3.4.6. Survivability

Failures can affect a large number of VN requests, which can be divided into two categories: node failure [77,78] and link failure [79]. VN survivability is the ability that a VN continuously provides services in compliance with the given requirements in present failures and other events [79,80]. Link survivability can be classified into two categories: protection [81,82] and restoration [83]. The authors of [84,85] have focused on the link recovery in C-RAN.

To solve the issue, the proposed survivable algorithms should consider some performance metrics as follows.

- Number of backups: The metric counts the number of backup resources that is reserved for a VN. Additional substrate resources have to be reserved to serve the VN request when failures happen. Path Redundancy measures the ratio between the number of backup paths to the number of direct paths. Some redundancy algorithms set up backup paths that can be used in case some parts of the network break down [86]. Therefore, the metric refers to the amount of additional resources that are used to backup the embedded network.
- Migration frequency: For node failure, migration frequency shows the performance required to achieve higher acceptance ratio and lower embedding cost of node migration [77]. The affected task node will be migrated to one backup host after node failure to reduce the cost of node migration and re-embedding the path. Link failure or path length constraint also can trigger migrations. Therefore, migration frequency should be considered as a metric to show the migration performance.

#### 3.4.7. Traffic Prediction

Traffic prediction is not an objective or a metric; however, it is an important procedure to improve objective or metric performance. In many 5G scenarios and applications, network traffic prediction is playing an important role [87] for resource allocation and load balancing in management and provisioning (e.g., network management, traffic (re)-routing). Autoregressive (AR), autoregressive moving average (ARMA), autoregressive integrated moving average (ARIMA), and support vector machine (SVM) are the most common models for network prediction problems [88]. Due to the ability of processing the high-dimensional data, deep/convolutional/recurrent neural network models are widely used in the network traffic prediction to improve the accuracy of prediction [88,89,90,91,92]. The accuracy of the predicted results based on the algorithms needs to guarantee the change of the bandwidth explosion and service diversity. In addition, the accuracy is used to measure how efficient VNE method impacts the number of VN requests or the profit ratio for InPs.

To evaluate the prediction accuracy, mean absolute error (MAE) [93], measure square error (MSE) [94], and root mean square errors (RMSE) [88] are used to quantify the difference between the forecasted values and the actual values. MAE is an average sum of the absolute errors described in Equation (14), where $y_{i}$ and $\hat{y_{i}}$ are the observed value and the predicted value, and *N* represents the total number of predictions. MAE is a widely used prediction accuracy measurement and a small value of it means that the predictor has high performance. MSE is a scale dependent metric by computing the average sum of squared errors as shown in Equation (15). In addition, RMSE is the square root of MSE as shown in Equation (16).

(14) $$MAE=\frac{1}{N}\sum_{i=1}^{N}\left|y_{i}-\hat{y_{i}}\right|$$

(15) $$MSE=\frac{1}{N}\sum_{i=1}^{N}{\left(y_{i}-\hat{y_{i}}\right)}^{2}$$

(16) $$RMSE=\sqrt{\frac{1}{N}\sum_{i=1}^{N}{\left(y_{i}-\hat{y_{i}}\right)}^{2}}$$

## 4. VNE Algorithms Taxonomy

According to the VNE problem description and formulation in Section 3, ILP with various objectives and constraints of network architecture features is an optimal VNE solution for many existing research works [11,14,69]. However, the VNE problem is known as an NP-hard problem, which cannot be solved in polynomial time when the substrate network topology or virtual network requests are scaled through ILP. To overcome the issue, many existing research works have proposed novel algorithms. The VNE problem is divided into two sub-problems as described in Section 3, we review the methods for VNoE and VLiE. In this section, we classify the existing VNE algorithms into three categories: two-stage VNE algorithms, coordinated VNE algorithms, and machine learning-based VNE algorithms.

### 4.1. Two-Stage VNE Algorithms

Two-stage VNE algorithms execute VNoE according to node ranking strategy for virtual nodes. The link assignment strategy of VLiE is executed for resource allocation after all the virtual nodes embedded. This section reviews the representative node ranking strategies for VNoE and virtual link assigment approaches for VLiE.

#### 4.1.1. Virtual Node Embedding

For virtual node embedding, greedy strategy is the most common method for virtual node resource allocation. The choice of method used in ranking the virtual node and substrate node efficiently is essential for embedding. The most considered metric for node ranking is the CPU capacity in decreasing order according to the requirement of virtual nodes and residual CPU capacity of substrate nodes [76]. Available resource node ranking method is formulated as product of node residual CPU capacity and sum of unoccupied bandwidth capacity of the neighbor links [12,95,96]. Instead of CPU capacity, the available resource method ensures that enough CPU capacity available and also considers bandwidth capacity to prepare for the subsequent link mapping stage. The authors of [97] propose a candidate-assisted algorithm by constructing candidate substrate nodes and candidate substrate paths for a virtual network request to reduce the mapping execution time.

The Markov random walk (RW) topology-aware node ranking method is proposed inspired by the PageRank algorithm [63], which is computed using a classic iterative scheme for the product of the vector of all substrate nodes resource and a one-step stochastic transition matrix of the Markov chain. To quantify the embedding potential of each node in the substrate network, the proposed global resource capacity (GRC) takes the topological attributes resources of the entire network into consideration [15]. The GRC value for each node is the sum of weighted normalized residual CPU capacity and bandwidth resource of links connected with the node. GRC node rankings are computed using an iterative scheme of the GRC vector which is composed of the calculated GRC for the nodes. An additional attribute is considered in [69,98] for the formulated modified GRC method.

#### 4.1.2. Virtual Link Embedding

After all nodes embedded in VNoE, virtual link embedding strategy is executed as fixed source–destination link requirement assignment. The most common used strategies for wired network are Dijkstra shortest path (SP) [76,95,98], K-shortest path (KSP) [12,14,36,63], and multi-commodity flow (MCF) [99]. The authors of [36] have considered link availability for survivability. Additional link restrictions of network (e.g., spectrum continuity, spectrum contiguity, and modulations) need to be considered. The authors of [12] have considered path splitting to reduce link congestion for better link resource allocation. The authors of [96,97] have proposed to construct satisfied the virtual link requirements candidate path set with reduction of mapping execution time, meanwhile without loss of the main performance indices.

### 4.2. Coordinated VNE Algorithms

Coordinated VNE approaches have been proposed to improve network resource utilization and solve the drawback and limitation of two-stage VNE approaches that it may cause embedding failure due to inefficient link capacity after all vitrtual node embedded. In [15,99], the proposed coordinated VNE algorithms consider node and link stage jointly to improve the performance metrics. The proposed coordinated VNE algorithms execute virtual link embedding according to descending order of the degree of virtual node for the virtual link [57]. Additional link restrictions (e.g., spectrum continuity, spectrum contiguity, and modulations) have been considered in EON with multi-core fiber. Due to the unique features of wireless networks, SP or MCF is not appropriate for mapping. Taking advantage of the broadcast nature of wireless channels, authors have proposed an anypath link mapping scheme to fulfill the diverse QoS requirements of VNs and reduce the resources consumed by retransmissions [32].

In order to further improve the performance, metaheuristic-based coordinated VNE algorithms are proposed. The authors of [48] propose an effective genetic algorithm for virtual optical network mapping, core allocation, and spectrum assignment in EONs using multi-core fibers. The objective is minimizing the maximum index of used frequency slots, which is regard as spectrum usage in this section. Virtual nodes mapping population, routing population, and core allocation population are used in proposed genetic algorithm. A periodical planning of embedding process is proposed in [100], where profitable VN requests are selected through an auction mechanism to maximize the revenue. The authors of [56,69] have proposed ant colony optimization (ACO)-based VNE algorithm in inter-ODCN. The proposed multi-objectives VNE algorithm based on particle swarm optimization (PSO) in [101] can improve the energy and revenue performance through the particle iteration.

### 4.3. Machine Learning Based VNE Algorithms

Two-stage and coordinated VNE algorithms will lead to a sub-optimal solution because of artificial rules. Due to the development of ML [102,103] technologies, efficient VNE algorithms based on ML have received greater concern in satisfying the increasing diversity of applications and demands and to reduce search space. The authors of [104] formalize the virtual node mapping problem by using the Markov decision process (MDP) framework and devise node mappings for the proposed MDP using the Monte Carlo tree search algorithm.

Several works have appeared on the design of VNE solution using reinforcement learning (RL), which focuses on how to interact with the environment to achieve maximum cumulative return. The authors of [105] proposed a RL-based dynamic attribute matrix representation algorithm for VNE. Substrate network node information and link information are represented by an attribute matrix and an adjacency matrix. Furthermore, a novel approach, NeuroViNE, to speed up and improve the existing VNE algorithms has been proposed in [106], where NeuroViNE relies on Hopfield network to reduce search space and preprocess the problem. The Hopfield network is a form of recurrent neural network, which can extract whole valuable subgraphs and compute a probability for each node. The authors of [107] have developed a DRL-based VNE solution called DeepViNE. The key idea is to encode substrate and virtual networks as two-dimensional images.

In IWNs, the dynamic link quality and time-varying workload of the forwarding nodes make it intractable for the optimal anypath forwarding actions computation. To better learn the environment, deep Q-learning by combining deep neural network with Q-learning is used to solve VNE in IWNs [24]. Based on the above literature review, we summarize the representative references and list typical VNE algorithms in Table 1, arranged in different categories as given in section.

## 5. Issues and Challenges

Although VNE algorithms are currently undergoing a comprehensive research phase, there are still numerous challenges and research problems to be addressed, especially for ML-based algorithms. The existing works mainly focus on single network domain; furthermore, the VNE problem for multi-domain networks still challenge. Similar to other emerging technologies, network slicing brings forward a significant potential toward 5G, but introduces several technical and business challenges by regarding as architecture and deployment. In this section, we discuss the future challenges and experiences learned on the road of VNE approaches. Three main fields, but not limited to that, may be focused in the near future are identified: 5G architecture network slicing, field trial deployment, and ML-based approaches.

### 5.1. 5G Architecture Network Slicing

In comparison with the wireless network and optical network architectures, there are many challenges in the 5G multi-domain heterogeneous network (e.g., signal propagation, interference, user mobility, radio access technology, and optical signal). Network slicing towards 5G is envisioned to support multi-domain heterogeneous network with a widely range diverse set of performance and requirement services. Multi-domain 5G network orchestration has obtained more consideration in network slicing. Slicing the physical network into multiple isolated logical networks to support various VN requirements has emerged as a key solution to management the network resources. The authors of [35] have considered the user mobility in 5G network scenario. Some survey works for wireless network virtualization and 5G have been proposed [108,109], which have analyzed the state-of-the-art and challenges.

To implement and manage network slices, spectrum slicing problem and efficient bandwidth resources sharing among different slices should be solved according to the requirements. To solve this issue, the authors of [110] have presented a prototype in the C-RAN using Open Air Interface platform and SDN controller to validate the feasibility of configuring multiple slices on demands. According to the presented documentation by organizations such as ETSI, the network slice manager needs to follow the following features; services management, QoS, service composition, and service sharing. The network slice manager has been developed and validated in the multimedia real-time communications over optical network considering two network slices with different QoS [111].

In addition, due to the connectivity guarantee of the heterogeneous characteristics of the IoT ubiquitous network, resource allocation and energy efficiency improvement are challenging for the 5G scenario. Intelligent VNE for IWNs pervasive devices with sensing, processing, and connecting capabilities has been described in [24]. Furthermore, many 5G scenarios by regarding as IoV need to be addressed and discussed in the future.

### 5.2. Field Trial Deployment

As many researchers have focused on the network slicing and VNE approaches by simulation [4,24,112], how to evaluate the network performance using tools and experiment is one of the challenges. Net2Plan (http://www.net2plan.com/) is an open source Java-based network planning optimization software tool, which is designed with the aim to overcome the barriers imposed by existing network planning tools to integrate customized algorithm of users. Net2Plan can define a network representation, based on abstract concepts such as nodes, links, traffic demands, routes, protection segments, shared-risk groups, and network layers [113,114]. The authors of [115] have demonstrated an open source Net2Plan extension interfacing multiple OpenStack instances for enabling multi-datacenter IT resource management, with multi-tenant slicing in an ETSI-OSM orchestrated and ONOS-controlled IP over WDM transport network. However, intelligent functions and modules are still need to be addressed in the future works for the deployment tools.

As the growing of some enabling techniques (e.g., SDN), the deployment of network virtualization has obtained more considerations [26,27], where OpenDaylight and OpenStack are the most common tools to establish the platform for resource provisioning and demonstration [116,117,118,119]. To offer the implementation of network virtualization according to the diverse requirements of applications, SDN is an ideal platform through hypervisors such as Flowvisor, OpenVirteX, FlowN, and AutoVFlow [24]. The authors of [120] have focused on implementation of automatic network slicing for microservices, where open source software Node-RED is modified and extended to design IoT services for implementation. Open Air Interface platform and FlexRan controller are used for network slicing implementation of C-RAN for eMBB and IoT slices [110,121]. For vertical services slicing and orchestration solutions in 5G infrastructures, eMBB network slices instantiated interconnecting physical and virtual functions, provisioned and configured on-demand have been proposed in [122]. The authors of [123] have described a programmable optical software-defined network testbed, which has been upgraded to offer backhaul and fronthaul transport capabilities in support of C-RAN functionalities with increased reliability. For the inter elastic ODCNs domain, proposed feasible virtualized bandwidth variable transceiver (V-BVT) architecture for network slicing implementation has been demonstrated by an experimental platform with SDN controller to maintain the coexisting and isolation features in the physical layer in [124,125].

To implement and valid slicing in 5G networks, researchers should keep their eyes on the implementation technologies and devices. Field trial deployment for network slicing on multi-domain heterogeneous 5G architectures to support 5G services still have many challenges and should obtain more concerns.

### 5.3. Machine Learning Based Management Algorithm

Due to high-bandwidth and low-latency applications increasing the burden of network, network management and resource allocation need more dynamic and self-adaptive approaches to address the problem. VNE has obtained more concerns due to its importance for 5G network slicing. Some existing works [106,107] have addressed the issues by considering ML technique to learn how to allocate resource and manage the service request itself automatically. However, more ML-based algorithms for VNE need to be proposed in the future to obtain better performance metrics (e.g., profit, latency, energy efficiency, and survivability) for network management dynamically [126].

To provide automatic embedding solutions, the authors of [127] have proposed a novel algorithm combining reinforcement learning with a novel neural network structure for general network. In [128], the authors have proposed an efficient VNE algorithm adopting parallel reinforcement learning framework with graph convolutional network. Asynchronous advantage actor–critic-based policy gradient method is selected to train the network parameters. Simulation results of the proposed algorithm outperforms the typical VNE algorithms. However, majority latest ML-based VNE algorithms are based on the general network architecture without consideration additional characteristics such as optical network nodes and links. The generality of ML-based VNE algorithms should obtain more consideration for multi-domain heterogeneous network architecture.

In addition, the analysis of traffic demands can reveal valuable information for the management to gather information interacting with SDN to centralize control network. Traffic prediction strategy is essential to predict future traffic matrix via its prior measurements, where services can be provisioned taking into account future resource needs [129]. Some existing works have focused on ML-based traffic prediction strategies [130,131]. Recurrent neural networks have been designed for sequence prediction problem to optimize resource allocation of optical backbone network, where gate recurrent units (GRU) in RNN have been considered to achieve great accuracy [93]. Convolutional neural network (CNN) and long short-term memory (LSTM) are integrated for modeling and estimating the future network traffic [131]. Furthermore, ML-based traffic prediction mechanisms for VNE need to be proposed in the future.

## 6. Conclusions

This paper has presented a survey of existing works on the VNE problem towards multi-domain heterogeneous converged optical network, which have focused on the resource allocation optimization of multiple virtual networks coexisting and sharing resource in substrate networks. We have pointed out the features of the multi-domain heterogeneous 5G network architectures, where special constraints have to be considered for VNE according to the features of various network architecture (e.g., wireless network). The basic VNE problem with motivation and performance metrics has been described in details for general network scenario. A VNE algorithm taxonomy has been proposed for analyzing the existing VNE algorithms according to two-stage, coordinated, and machine learning-based algorithms. We have analyzed the issues and challenges of VNE towards multi-domain heterogeneous network, and pointed out some promising research directions: 5G architecture network slicing and field trail deployment for VNE- and ML-based management algorithms for resource allocation.

## Figures and Tables

**Table 1:** VNE algorithm taxonomy.

Algorithm	Network	Request Types	Objectives	Network Control	ILP	Node Ranking	Link Assignment	Reference
Two-stage	General	Dynamic	Revenue	N	Y	Available resource	KSP + splitting	[12] Yu et al. (2008)
					N	RW	KSP	[63] Zhang et al. (2012)
			Cost	N	Y	Candidate node set	Candidate path set	[97] Cao et al. (2018)
			Energy efficiency	N	Y	Residual CPU	SP	[76] Zhang et al. (2016)
						Modified GRC	SP	[98] Cao et al. (2018)
	FiWi	Static	Survivablility	N	Y	Residual CPU	KSP	[36] Liu et al. (2019)
	Inter—ODCN	Dynamic	Cost	N	Y	Available resource	SP	[95] Jiang et al. (2015)
			Acceptance	Y	Y	Available resource	Candidate path set	[96]Pagès et al. (2019)
	EON	Static	Spectrum usage	N	Y	Random	KSP + splitting	[14] Shahriar et al. (2019)
Coordinated	General	Dynamic	Revenue	N	N	GRC	SP	[15] Gong et al. (2014)
			Cost	N	Y	Available resource	MCF + splitting	[99] Chowdhury et al. (2012)
			Energy efficiency+ Revenue	N	N	Candidate node set	SP	[101] Shahin et al. (2015)
			Revenue	N	Y	N/A	N/A	[100] Jarray et al. (2015)
	WSN	Dynamic	Revenue	N	N	N/A	anypath	[32] Li et al. (2017)
	EON	Static	Cost	N	Y	N/A	SP	[57] Lin et al. (2018)
			Spectrum usage	N	N	Random	KSP	[48] Xuan et al. (2017)
	Inter—ODCN	Static	Energy efficiency	Y	Y	Modified GRC	SP	[69] Zong et al. (2018)
		Dynamic	Acceptance	Y	Y	Residual CPU	SP	[56] Fajjari et al. (2014)
ML	IWN	Static	Latency	Y	N	N/A	Anypath	[24] Li et al. (2019)
	General	Dynamic	Revenue + cost	N	N	Residual CPU	N/A	[106] Blenk et al. (2018)
						N/A	N/A	[107] Dolati et al. (2019)
			Profit	N	N	MCTS	MCF	[104] Haeri et al. (2018)

## References

[1] Ordonez-Lucena J., Ameigeiras P., Lopez D., Ramos-Munoz J.J., Lorca J., Folgueira J. (2017). Network Slicing for 5G with SDN/NFV: Concepts, Architectures, and Challenges. IEEE Commun. Mag..

[2] Foukas X., Patounas G., Elmokashfi A., Marina M.K. (2017). Network Slicing in 5G: Survey and Challenges. IEEE Commun. Mag..

[3] Zhang S. (2019). An Overview of Network Slicing for 5G. IEEE Wirel. Commun..

[4] Addad R., Bagaa M., Taleb T., Cadette Dutra D.L., Flinck H. (2019). Optimization Model for Cross-Domain Network Slices in 5G Networks. IEEE Trans. Mob. Comput..

[5] Afolabi I., Taleb T., Samdanis K., Ksentini A., Flinck H. (2018). Network Slicing and Softwarization: A Survey on Principles, Enabling Technologies, and Solutions. IEEE Commun. Surv. Tutorials.

[6] Bizanis N., Kuipers F.A. (2016). SDN and Virtualization Solutions for the Internet of Things: A Survey. IEEE Access.

[7] BinSahaq A., Sheltami T., Salah K. (2019). A Survey on Autonomic Provisioning and Management of QoS in SDN Networks. IEEE Access.

[8] Wang A., Iyer M., Dutta R., Rouskas G.N., Baldine I. (2013). Network Virtualization: Technologies, Perspectives, and Frontiers. J. Lightwave Technol..

[9] Chowdhury N.M.M.K., Boutaba R. (2009). Network Virtualization: State of the Art and Research Challenges. IEEE Commun. Mag..

[10] Chowdhury N.M.M.K., Boutaba R. (2010). A Survey of Network Virtualization. Comput. Netw..

[11] Wang Y., McNulty Z., Nguyen H. (2017). Network Virtualization in Spectrum Sliced Elastic Optical Path Networks. J. Lightwave Technol..

[12] Yu M., Yi Y., Rexford J., Chiang M. (2008). Rethinking Virtual Network Embedding: Substrate Support for Path Splitting and Migration. ACM SIGCOMM Comput. Commun. Rev..

[13] Cheng X., Su S., Zhang Z., Shuang K., Yang F., Luo Y., Wang J. (2012). Virtual Network Embedding through Topology Awareness and Optimization. Comput. Netw..

[14] Shahriar N., Taeb S., Chowdhury S.R., Tornatore M., Boutaba R., Mitra J., Hemmati M. Achieving a Fully-Flexible Virtual Network Embedding in Elastic Optical Networks. Proceedings of the IEEE INFOCOM 2019—IEEE Conference on Computer Communications.

[15] Gong L., Wen Y., Zhu Z., Lee T. Toward Profit-seeking Virtual Network Embedding Algorithm via Global Resource Capacity. Proceedings of the IEEE INFOCOM 2014 - IEEE Conference on Computer Communications.

[16] Davalos E.J., Baran B. (2018). A Survey on Algorithmic Aspects of Virtual Optical Network Embedding for Cloud Networks. IEEE Access.

[17] Bari M.F., Boutaba R., Esteves R., Granville L.Z., Podlesny M., Rabbani M.G., Zhang Q., Zhani M.F. (2013). Data Center Network Virtualization: A Survey. IEEE Commun. Surv. Tutor..

[18] Singh S., Jeong Y.S., Park J.H. (2016). A Survey on Cloud Computing Security: Issues, Threats, and Solutions. J. Netw. Comput. Appl..

[19] Taleb T., Afolabi I., Bagaa M. (2019). Orchestrating 5G Network Slices to Support Industrial Internet and to Shape Next-Generation Smart Factories. IEEE Netw..

[20] Zhou Y., Yin S., Guo B., Huang H., Li W., Zhang M., Huang S. (2016). Experimental Demonstration of Software-Defined Optical Network for Heterogeneous Packet and Optical Networks. Photonic Netw. Commun..

[21] Zhou Y., Ramamurthy B., Guo B., Huang S. (2018). Supporting Dynamic Bandwidth Adjustment Based on Virtual Transport Link in Software-Defined IP Over Optical Networks. J. Opt. Commun. Netw..

[22] Yang H., Zhang J., Zhao Y., Li H., Huang S., Ji Y., Han J., Lin Y., Lee Y. (2014). Cross Stratum Resilience for OpenFlow-enabled Data Center Interconnection with Flexi-Grid Optical Networks. Opt. Switch. Netw..

[23] Han Y., Hyun J., Hong J.W.K. Graph Abstraction based Virtual Network Management Framework for SDN. Proceedings of the 2016 IEEE Conference on Computer Communications Workshops (INFOCOM WKSHPS).

[24] Li M., Chen C., Hua C., Guan X. (2019). Intelligent Latency-Aware Virtual Network Embedding for Industrial Wireless Networks. IEEE IoT J..

[25] Medhat A.M., Taleb T., Elmangoush A., Carella G.A., Covaci S., Magedanz T. (2017). Service Function Chaining in Next Generation Networks: State of the Art and Research Challenges. IEEE Commun. Mag..

[26] Hammad A., Aguado A., Peng S., Vilalta R., Mayoral A., Casellas R., Martínez R., Muñoz R., Nejabati R., Simeonidou D. On-demand Virtual Infrastructure Composition over Multi- domain and Multi-technology Networks. Proceedings of the IEEE Optical Fiber Communications Conference and Exhibition (OFC).

[27] Martínez R., Mayoral A., Vilalta R., Casellas R., Muñoz R., Pachnicke S., Szyrkowiec T., Autenrieth A. (2017). Integrated SDN/NFV Orchestration for the Dynamic Deployment of Mobile Virtual Backhaul Networks Over a Multilayer (Packet/Optical) Aggregation Infrastructure. J. Opt. Commun. Netw..

[28] Wang X., Cavdar C., Wang L., Tornatore M., Zhao Y., Chung H., Lee H.H., Park S., Mukherjee B. Joint Allocation of Radio and Optical Resources in Virtualized Cloud RAN with CoMP. Proceedings of the 2016 IEEE Global Communications Conference (GLOBECOM).

[29] Hu Y.C., Patel M., Sabella D., Sprecher N., Young V. (2015). Mobile Edge Computing—A key technology towards 5G. ETSI White Pap..

[30] Abbas N., Zhang Y., Taherkordi A., Skeie T. (2018). Mobile Edge Computing: A Survey. IEEE IoT J..

[31] Khan I., Belqasmi F., Glitho R., Crespi N., Morrow M., Polakos P. (2016). Wireless Sensor Network Virtualization: A Survey. IEEE Commun. Surv. Tutor..

[32] Li M., Hua C., Chen C., Guan X. Application-driven Virtual Network Embedding for Industrial Wireless Sensor Networks. Proceedings of the 2017 IEEE International Conference on Communications (ICC).

[33] Yun D., Ok J., Shin B., Park S., Yi Y. (2013). Embedding of Virtual Network Requests over Static Wireless Multihop Networks. Comput. Netw..

[34] Lv P., Wang X., Xu M. (2012). Virtual Access Network Embedding in Wireless Mesh Networks. Ad Hoc Netw..

[35] Guan Y., Zong Y., Liu Y., Guo L., Ning Z., Rodrigues J.J.P.C. Virtual Network Embedding Supporting User Mobility in 5G Metro/Access Networks. Proceedings of the ICC 2019—2019 IEEE International Conference on Communications (ICC).

[36] Liu Y., Han P., Hou J., Zheng J. (2019). Resource-Efficiently Survivable IoT Services Provisioning via Virtual Network Embedding in Fiber-Wireless Access Network. IEEE Access.

[37] Han P., Liu Y., Guo L. (2018). QoS Satisfaction Aware and Network Reconfiguration Enabled Resource Allocation for Virtual Network Embedding in Fiber-Wireless Access Network. Comput. Netw..

[38] Wang W., Guo W., Hu W. Network Service Slicing Supporting Ubiquitous Access in Passive Optical Networks. Proceedings of the 2018 20th International Conference on Transparent Optical Networks (ICTON).

[39] Han P., Guo L., Liu Y. Virtual Network Embedding in SDN/NFV based Fiber-Wireless Access Network. Proceedings of the International Conference on Software Networking.

[40] Mosahebfard M., Vardakas J., Ramantas K., Verikoukis C. SDN/NFV-based Network Resource Management for Converged Optical-wireless Network Architectures. Proceedings of the 2019 21st International Conference on Transparent Optical Networks (ICTON).

[41] Wang Q., Shou G., Liu J., Liu Y., Hu Y., Guo Z. (2019). Resource Allocation for Edge Computing over Fibre-wireless Access Networks. IET Commun..

[42] Rahman S., Gupta A., Tomatore M., Mukherjee B. (2017). Dynamic Workload Migration over Optical Backbone Network to Minimize Data Center Electricity Cost. IEEE Trans. Green Commun. Netw..

[43] Zhang J., Ji Y., Song M., Li H., Gu R., Zhao Y., Zhang J. (2015). Dynamic Virtual Network Embedding over Multilayer Optical Networks. J. Opt. Commun. Netw..

[44] Rodriguez E., Alkmim G.P., Da Fonseca N.L., Batista D.M. (2017). Energy-Aware Mapping and Live Migration of Virtual Networks. IEEE Syst. J..

[45] Taeb S., Shahriar N., Chowdhury S.R., Tornatore M., Boutaba R. Virtual Network Embedding with Path-based Latency Guarantees in Elastic Optical Networks. Proceedings of the 2019 IEEE 27th International Conference on Network Protocols (ICNP).

[46] Huang S., Zhou Y., Yin S., Kong Q., Zhang M., Zhao Y., Zhang J., Gu W. (2014). Fragmentation Assessment based On-line Routing and Spectrum Allocation for Intra-data-center Networks with Centralized Control. Opt. Switch. Netw..

[47] Klonidis D., Cugini F., Gerstel O., Jinno M., Lopez V., Palkopoulou E., Sekiya M., Siracusa D., Thouénon G., Betoule C. (2015). Spectrally and Spatially Flexible Optical Network Planning and Operations. IEEE Commun. Mag..

[48] Xuan H., Wang Y., Xu Z., Hao S., Wang X. (2017). Virtual Optical Network Mapping and Core Allocation in Elastic Optical Networks using Multi-Core Fibers. Opt. Commun..

[49] Huang H., Huang S., Yin S., Zhang M., Zhang J., Gu W. (2016). Virtual Network Provisioning Over Space Division Multiplexed Optical Networks Using Few-Mode Fibers. J. Opt. Commun. Netw..

[50] Ou Y., Hammad A., Peng S., Nejabati R., Simeonidou D. (2015). Online and Offline Virtualization of Optical Transceiver. J. Opt. Commun. Netw..

[51] Li X., Zhang L., Tang Y., Gao T., Zhang Y., Huang S. (2018). On-demand Routing, Modulation Level and Spectrum Allocation (OD-RMSA) for Multicast Service Aggregation in Elastic Optical Networks. Opt. Express.

[52] Gao T., Li X., Guo B., Yin S., Li W., Huang S. (2017). Spectrum-efficient Multipath Provisioning with Content Connectivity for the Survivability of Elastic Optical Datacenter Networks. Opt. Fiber Technol..

[53] Guo B., Shang Y., Zhang Y., Li W., Yin S., Zhang Y., Huang S. (2019). Timeslot Switching-Based Optical Bypass in Data Center for Intrarack Elephant Flow with an Ultrafast DPDK-Enabled Timeslot Allocator. J. Lightwave Technol..

[54] Li X., Zhang L., Tang Y., Guo J., Huang S. (2018). Distributed Sub-Tree-Based Optical Multicasting Scheme in Elastic Optical Data Center Networks. IEEE Access.

[55] Li X., Yin S., Wang X., Zhou Y., Zhao Y., Huang S., Zhang J. (2017). Content Placement With Maximum Number of End-to-Content Paths in k-Node (Edge) Content Connected Optical Datacenter Networks. J. Opt. Commun. Netw..

[56] Fajjari I., Aitsaadi N., Pióro M., Pujolle G. (2014). A New Virtual Network Static Embedding Strategy within the Cloud’s Private Backbone Network. Comput. Netw..

[57] Lin R., Luo S., Zhou J., Wang S., Cai A., Zhong W., Zukerman M. (2018). Virtual Network Embedding with Adaptive Modulation in Flexi-Grid Networks. J. Lightwave Technol..

[58] Huang H., Guo B., Li X., Yin S., Zhou Y., Huang S. (2017). Crosstalk-aware Virtual Network Embedding over Inter-datacenter Optical Networks with Few-mode Fibers. Opt. Fiber Technol..

[59] Zhu M., Zhang S., Sun Q., Li G., Chen B., Gu J. (2018). Fragmentation-aware VONE in elastic optical networks. J. Opt. Commun. Netw..

[60] Montero R., Agraz F., Pages A., Spadaro S. End-to-End 5G Service Deployment and Orchestration in Optical Networks with QoE Guarantees. Proceedings of the 2018 20th International Conference on Transparent Optical Networks (ICTON).

[61] Cao H., Yang L., Zhu H. (2018). Novel Node-Ranking Approach and Multiple Topology Attributes-Based Embedding Algorithm for Single-Domain Virtual Network Embedding. IEEE IoT J..

[62] Chochlidakis G., Friderikos V. (2017). Mobility Aware Virtual Network Embedding. IEEE Trans. Mob. Comput..

[63] Zhang S., Qian Z., Wu J., Lu S. An Opportunistic Resource Sharing and Topology-aware Mapping Framework for Virtual Networks. Proceedings of the 2012 IEEE INFOCOM.

[64] Chen T., Liu J., Tang Q., Huang T., Huo R. (2019). Virtual Network Embedding Algorithm for Location-Based Identifier Allocation. IEEE Access.

[65] Cheng X., Su S., Zhang Z., Wang H., Yang F., Luo Y., Wang J. (2011). Virtual Network Embedding through Topology-aware Node Ranking. ACM SIGCOMM Comput. Commun. Rev..

[66] Zhu M., Sun Q., Zhang S., Gao P., Chen B., Gu J. (2019). Energy-Aware Virtual Optical Network Embedding in Sliceable- Transponder- Enabled Elastic Optical Networks. IEEE Access.

[67] Ning Z., Huang J., Wang X., Rodrigues J.J.P.C., Guo L. (2019). Mobile Edge Computing-Enabled Internet of Vehicles: Toward Energy-Efficient Scheduling. IEEE Netw..

[68] Chochlidakis G., Friderikos V. Low Latency Virtual Network Embedding for Mobile Networks. Proceedings of the 2016 IEEE International Conference on Communications (ICC).

[69] Zong Y., Ou Y., Hammad A., Kondepu K., Nejabati R., Simeonidou D., Liu Y., Guo L. (2018). Location-Aware Energy Efficient Virtual Network Embedding in Software-Defined Optical Data Center Networks. J. Opt. Commun. Netw..

[70] Hejja K., Hesselbach X. (2018). Online Power Aware Coordinated Virtual Network Embedding with 5G Delay Constraint. J. Netw. Comput. Appl..

[71] Song C., Zhang M., Zhan Y., Wang D., Guan L., Liu W., Zhang L., Xu S. (2019). Hierarchical Edge Cloud Enabling Network Slicing for 5G Optical Fronthaul. J. Opt. Commun. Netw..

[72] Nonde L., Elgorashi T.E., Elmirgahni J.M. Virtual Network Embedding Employing Renewable Energy Sources. Proceedings of the 2016 IEEE Global Communications Conference (GLOBECOM).

[73] Zhu M., Gao P., Zhang J., Zeng X., Zhang S. Energy Efficient Dynamic Virtual Optical Network Embedding in Sliceable-Transponder-Equipped EONs. Proceedings of the GLOBECOM 2017—2017 IEEE Global Communications Conference.

[74] Nonde L., El-gorashi T.E.H., Elmirghani J.M.H. (2015). Energy Efficient Virtual Network Embedding for Cloud Networks. J. Lightwave Technol..

[75] Xiong Y., Shi J., Yang Y., Lv Y., Rouskas G.N. (2018). Lightpath Management in SDN-Based Elastic Optical Networks with Power Consumption Considerations. J. Lightwave Technol..

[76] Zhang Z., Su S., Shuang K., Li W., Zia M.A. Energy Aware Virtual Network Migration. Proceedings of the GLOBECOM 2016—2016 IEEE Global Communications Conference.

[77] Guo B., Qiao C., Wang J., Yu H., Zuo Y., Li J., Chen Z., He Y. (2014). Survivable Virtual Network Design and Embedding to Survive a Facility Node Failure. J. Lightwave Technol..

[78] Li X., Gao T., Zhang L., Tang Y., Zhang Y., Huang S. (2018). Survivable K-Node (Edge) Content Connected Virtual Optical Network (KC-VON) Embedding Over Elastic Optical Data Center Networks. IEEE Access.

[79] Su Y., Meng X., Kang Q., Han X. (2018). Survivable Virtual Network Link Protection Method Based on Network Coding and Protection Circuit. IEEE Access.

[80] Khan A., An X., Iwashina S. (2016). Virtual Network Embedding for telco-grade Network Protection and Service Availability. Comput. Commun..

[81] Chowdhury S.R., Ahmed R., Khan M.M.A., Shahriar N., Boutaba R., Mitra J., Zeng F. (2016). Dedicated Protection for Survivable Virtual Network Embedding. IEEE Trans. Netw. Serv. Manag..

[82] Ayoubi S., Chen Y., Assi C. (2016). Towards Promoting Backup-Sharing in Survivable Virtual Network Design. IEEE/ACM Trans. Netw..

[83] Aguado A., Davis M., Peng S., Álvarez M.V., López V., Szyrkowiec T., Autenrieth A., Vilalta R., Mayoral A., Muñoz R. (2016). Dynamic Virtual Network Reconfiguration over SDN Orchestrated Multitechnology Optical Transport Domains. J. Lightwave Technol..

[84] Kondepu K., Sgambelluri A., Sambo N., Giannone F., Castoldi P., Valcarenghi L. (2018). Orchestrating Lightpath Recovery and Flexible Functional Split to Preserve Virtualized RAN Connectivity. J. Opt. Commun. Netw..

[85] Ramanathan S., Kondepu K., Mirkhanzadeh B., Razo M., Tacca M., Valcarenghi L., Fumagalli A. Performance Evaluation of Two Service Recovery Strategies in Cloud-Native Radio Access Networks. Proceedings of the 2019 21st International Conference on Transparent Optical Networks (ICTON).

[86] Gao T., Zou W., Li X., Guo B., Huang S., Mukherjee B. (2019). Distributed Sub-light-tree based Multicast Provisioning with Shared Protection in Elastic Optical Datacenter Networks. Opt. Switch. Netw..

[87] Andreoletti D., Troia S., Musumeci F., Giordano S., Maier G., Tornatore M. Network Traffic Prediction based on Diffusion Convolutional Recurrent Neural Networks. Proceedings of the IEEE INFOCOM 2019—IEEE Conference on Computer Communications Workshops (INFOCOM WKSHPS).

[88] Cao X., Zhong Y., Zhou Y., Wang J., Zhu C., Zhang W. (2017). Interactive Temporal Recurrent Convolution Network for Traffic Prediction in Data Centers. IEEE Access.

[89] Singh S.K., Jukan A. (2018). Machine-Learning-Based Prediction for Resource (Re)allocation in Optical Data Center Networks. J. Opt. Commun. Netw..

[90] Mata J., de Miguel I., Durán R.J., Merayo N., Singh S.K., Jukan A., Chamania M. (2018). Artificial Intelligence (AI) Methods in Optical Networks: A Comprehensive Survey. Opt. Switch. Netw..

[91] Li Y., Liu H., Yang W., Hu D., Wang X., Xu W. (2016). Predicting Inter-Data-Center Network Traffic Using Elephant Flow and Sublink Information. IEEE Trans. Netw. Serv. Manag..

[92] Fadlullah Z.M., Tang F., Mao B., oKato N., Akashi O., Inoue T., Mizutani K. (2017). State-of-the-Art Deep Learning: Evolving Machine Intelligence Toward Tomorrow’s Intelligent Network Traffic Control Systems. IEEE Commun. Surv. Tutor..

[93] Troia S., Alvizu R., Zhou Y., Maier G., Pattavina A. Deep Learning-Based Traffic Prediction for Network Optimization. Proceedings of the International Conference on Transparent Optical Networks (ICTON).

[94] Azzouni A., Pujolle G. NeuTM: A Neural Network-based Framework for Traffic Matrix Prediction in SDN. Proceedings of the NOMS 2018—2018 IEEE/IFIP Network Operations and Management Symposium.

[95] Jiang H., Wang Y., Gong L., Zhu Z. (2015). Availability-Aware Survivable Virtual Network Embedding in Optical Datacenter Networks. J. Opt. Commun. Netw..

[96] Pagès A., Agraz F., Montero R., Landi G., Capitani M., Gallico D., Biancani M., Nejabati R., Simeonidou D., Spadaro S. (2019). Orchestrating Virtual Slices in Data Centre Infrastructures with Optical DCN. Opt. Fiber Technol..

[97] Cao H., Zhu Y., Zheng G., Yang L. (2018). A Novel Optimal Mapping Algorithm with Less Computational Complexity for Virtual Network Embedding. IEEE Trans. Netw. Serv. Manag..

[98] Cao H., Guo Y., Qu Z., Wu S., Zhu H., Yang L. (2018). ER-VNE: A Joint Energy and Revenue Embedding Algorithm for Embedding Virtual Networks. IEEE Access.

[99] Chowdhury M., Rahman M.R., Boutaba R. (2012). ViNEYard: Virtual Network Embedding Algorithms with Coordinated Node and Link Mapping. IEEE/ACM Trans. Netw..

[100] Jarray A., Karmouch A. (2015). Decomposition Approaches for Virtual Network Embedding with One-shot Node and Link mapping. IEEE/ACM Trans. Netw..

[101] Shahin A.A. (2015). Memetic Multi-Objective Particle Swarm Optimization-Based Energy-Aware Virtual Network Embedding. Int. J. Adv. Comput. Sci. Appl..

[102] Li R., Zhao Z., Sun Q., Chih-Lin I., Yang C., Chen X., Zhao M., Zhang H. (2018). Deep Reinforcement Learning for Resource Management in Network Slicing. IEEE Access.

[103] Sun G., Zemuy G.T., Xiong K. Dynamic Reservation and Deep Reinforcement Learning based Autonomous Resource Management for Wireless Virtual Networks. Proceedings of the International Performance Computing and Communications Conference (IPCCC).

[104] Haeri S., Trajković L. (2018). Virtual Network Embedding via Monte Carlo Tree Search. IEEE Trans. Cybern..

[105] Yao H., Zhang B., Zhang P., Wu S., Jiang C., Guo S. (2018). RDAM: A Reinforcement Learning Based Dynamic Attribute Matrix Representation for Virtual Network Embedding. IEEE Trans. Emerg. Top. Comput..

[106] Blenk A., Kalmbach P., Zerwas J., Jarschel M., Schmid S., Kellerer W. NeuroViNE: A Neural Preprocessor for Your Virtual Network Embedding Algorithm. Proceedings of the IEEE INFOCOM 2018—IEEE Conference on Computer Communications.

[107] Dolati M., Hassanpour S.B., Ghaderi M., Khonsari A. DeepViNE: Virtual Network Embedding with Deep Reinforcement Learning. Proceedings of the IEEE INFOCOM 2019—IEEE Conference on Computer Communications Workshops (INFOCOM WKSHPS).

[108] Kitindi E.J., Fu S., Jia Y., Kabir A., Wang Y. (2017). Wireless Network Virtualization with SDN and C-RAN for 5G Networks: Requirements, Opportunities, and Challenges. IEEE Access.

[109] Liang C., Yu F.R. (2015). Wireless Network Virtualization: A Survey, Some Research Issues and Challenges. IEEE Commun. Surv. Tutor..

[110] Costanzo S., Fajjari I., Aitsaadi N., Langar R. DEMO: SDN-based network slicing in C-RAN. Proceedings of the 2018 15th IEEE Annual Consumer Communications and Networking Conference (CCNC).

[111] Alemany P., Vilalta R., De La Cruz J.L., Pol A., Román A., Casellas R., Martínez R., Muñoz R. Experimental Validation of Network Slicing Management for Vertical Applications on Multimedia Real-time Communications over a Packet/optical Network. Proceedings of the 2019 21st International Conference on Transparent Optical Networks (ICTON).

[112] Zhao C., Parhami B. (2019). Virtual Network Embedding Through Graph Eigenspace Alignment. IEEE Trans. Netw. Serv. Manag..

[113] Pavon-Marino P., Izquierdo-Zaragoza J.L. (2015). Net2plan: An Open Source Network Planning Tool for Bridging the Gap between Academia and Industry. IEEE Netw..

[114] Romero-Gazquez J.L., Bueno-Delgado M.V., Moreno-Muro F.J., Pavon-Marino P. Net2plan-GIS: An Open-Source Net2Plan Extension Integrating GIS Data for 5G Network Planning. Proceedings of the 2018 20th International Conference on Transparent Optical Networks (ICTON).

[115] Garrich M., Hernández-Bastida M., San-Nicolás-Martínez C., Moreno-Muro F.J., Pavon-Marino P. (2019). The Net2Plan-OpenStack Project: IT Resource Manager for Metropolitan SDN/NFV Ecosystems.

[116] Szyrkowiec T., Autenrieth A., Gunning P., Wright P., Lord A., Elbers J.-P., Lumb A. (2014). First Field Demonstration of Cloud Datacenter Workflow Automation Employing Dynamic Optical Transport Network Resources under OpenStack and OpenFlow Orchestration. Opt. Express.

[117] Yang H., Zhang J., Ji Y., Tan Y., Lin Y., Han J., Lee Y. Data Center Service Locationlization based on Virtual Resource Migration in software Defined Elastic Optical Network. Proceedings of the IEEE Optical Fiber Communications Conference and Exhibition (OFC).

[118] Hammad A., Aguado A., Kondepu K., Zong Y., Marhuenda J., Yan S., Nejabati R., Simeonidou D. Demonstration of NFV Content Delivery using SDN- enabled Virtual Infrastructures. Proceedings of the IEEE Optical Fiber Communications Conference and Exhibition (OFC).

[119] Diallo T., Beldachi A.F., Muqaddas A.S., Silva R.S., Nejabati R., Tzanakaki A., Simeonidou D. Enabling Heterogenous Low Latency and High- bandwidth Virtual Network Services for 5G Utilizing a Flexible Optical Transport Network. Proceedings of the IEEE Optical Fiber Communications Conference and Exhibition (OFC).

[120] Minami Y., Taniguchi A., Kawabata T., Sakaida N., Shimano K. An Architecture and Implementation of Automatic Network Slicing for Microservices. Proceedings of the NOMS 2018 - 2018 IEEE/IFIP Network Operations and Management Symposium.

[121] Costanzo S., Cherrier S., Langar R. Network Slicing Orchestration of IoT-BeC3applications and eMBB services in C-RAN. Proceedings of the IEEE INFOCOM 2019—IEEE Conference on Computer Communications Workshops (INFOCOM WKSHPS).

[122] Landi G., Giardina P., Capitani M., Kondepu K., Valcarenghi L., Avino G. Provisioning and automated scaling of network slices for virtual Content Delivery Networks in 5G infrastructures. Proceedings of the ACM International Symposium on Mobile Ad Hoc Networking and Computing.

[123] Ramanathan S., Tacca M., Razo M., Mirkhanzadeh B., Kondepu K., Giannone F., Valcarenghi L., Fumagalli A. (2019). A programmable optical network testbed in support of C-RAN: a reliability study. Photonic Netw. Commun..

[124] Ou Y., Yan S., Hammad A., Guo B., Peng S., Nejabati R., Simeonidou D. (2016). Demonstration of Virtualizeable and Software-Defined Optical Transceiver. J. Lightwave Technol..

[125] Ou Y., Davis M., Aguado A., Meng F., Nejabati R., Simeonidou D. (2018). Optical Network Virtualisation Using Multitechnology Monitoring and SDN-Enabled Optical Transceiver. J. Lightwave Technol..

[126] Wang S., Bi J., Wu J., Vasilakos A.V., Fan Q. (2019). VNE-TD: A Virtual Network Embedding Algorithm Based on Temporal- Difference Learning. Comput. Netw..

[127] Yan Z., Ge J., Wu Y., Zheng H., Li L., Li T. Automatic Virtual Network Embedding based on Deep Reinforcement Learning. Proceedings of the IEEE International Conference on High Performance Computing and Communications, IEEE International Conference on Smart City and IEEE International Conference on Data Science and Systems.

[128] Yan Z., Ge J., Wu Y., Li L., Li T. (2020). Automatic Virtual Network Embedding: A Deep Reinforcement Learning Approach with Graph Convolutional Networks. IEEE J. Sel. Areas Commun..

[129] Zhang H., Zheng X., Tian J. Virtual Network Mapping based on the Prediction of Support Vector Machine. Proceedings of the 2016 8th International Conference on Information Technology in Medicine and Education (ITME).

[130] Alvizu R., Troia S., Maier G., Pattavina A. (2017). Matheuristic With Machine-Learning-Based Prediction for Software- Defined Mobile Metro-Core Networks. J. Opt. Commun. Netw..

[131] Le V.A., Nguyen P.L., Ji Y. Deep Convolutional LSTM Network-based Traffic Matrix Prediction with Partial Information. Proceedings of the 2019 IFIP/IEEE Symposium on Integrated Network and Service Management (IM).

